# Effect of the Interaction between Mental Stress and Eating Pattern on Body Mass Index Gain in Healthy Japanese Male Workers

**DOI:** 10.2188/jea.JE20080066

**Published:** 2009-03-19

**Authors:** Hideaki Toyoshima, Nobutaka Masuoka, Shuji Hashimoto, Rei Otsuka, Satoshi Sasaki, Koji Tamakoshi, Hiroshi Yatsuya

**Affiliations:** 1 Health Care Center, Anjo Kosei Hospital, Anjo, Aichi, Japan; 2 Department of Public Health/Health Information Dynamics, Nagoya University Graduate School of Medicine, Nagoya, Japan; 3 Department of Hygiene, Fujita Health University School of Medicine, Toyoake, Aichi, Japan; 4 Department of Epidemiology, National Institute for Longevity Sciences, Obu, Aichi, Japan; 5 Department of Social and Preventive Epidemiology, School of Public Health, the University of Tokyo, Tokyo, Japan; 6 Department of Nursing, Nagoya University School of Health Science, Nagoya, Japan

**Keywords:** mental stress, eating to satiety, interaction, body mass index gain, energy intake

## Abstract

**Background:**

The effect of the interaction between long-term mental stress and eating habits on weight gain has not been confirmed in humans.

**Methods:**

A population of 1080 healthy Japanese male local government employees without lifestyle-related diseases were studied. Height and weight were measured and perception of mental stress and the frequency of eating to satiety, drinking, smoking, and exercise were surveyed by means of a questionnaire in both 1997 and 2002. Exposure patterns during this 5-year period were classified as low or high. Information on daily food and energy intake was collected in 2002. The effect of the interaction between stress and the frequency of eating to satiety on change in BMI (ΔBMI) during this 5-year period was examined by 2-way analysis of variance (ANOVA) and covariance (ANCOVA) adjusted for age, BMI at baseline, and other lifestyle habits. The association between satiation eating and ΔBMI was compared between participants with high and low levels of stress.

**Results:**

Stress and satiation eating were not significantly mutually correlated. Two-way ANCOVA showed a significant interaction (F = 4.90, *P* = 0.03) between mental stress and satiation eating. Among participants with a high level of stress, BMI gain was significantly larger in those who ate to satiety than in those who ate moderately, when ΔBMI was unadjusted or adjusted for covariates (adjusted mean [SE]: 0.34 ± 0.06 kg/m^2^ vs. 0.12 ± 0.07 kg/m^2^, *P* = 0.002). Among participants with a low level of stress no such difference was observed. These results were unchanged after further adjustment for energy intake in 2002.

**Conclusion:**

In this population, eating pattern interacted with long-term mental stress to produce a larger body mass gain in satiation eaters than in moderate eaters among participants with a high level of stress, independent of energy intake or other lifestyle habits.

## INTRODUCTION

Abdominal obesity is a key factor in metabolic syndrome, and is linked with a number of life-threatening diseases. Various lifestyle factors are known to cause primary obesity.^1^ Although the effect of mental stress on obesity has been investigated in both humans^2^^–^^12^ and animals,^13^^–^^19^ the results vary depending on the species of animal studied, and on whether the mental stress was acute or chronic, participants were male or female, the stress was subjective or objective, and on whether the stressor was measured by a standardized method like the Karasek model^20^ or the Effort–Reward Imbalance Model.^21^

Recent animal experiments have indicated that the caloric efficiency of energy-dense food that is responsible for weight gain was higher in stressed, as compared to unstressed, rats due to the interaction of stress and food,^18^^,^^22^ and that stressed rats consumed larger proportions of “comfort food” containing lard and sugar.^22^ Although a growing number of studies that were done on the basis of an individual-difference model^8^ support the hypothesis that stress-induced eating eventually results in obesity, none has examined the effect of the interaction between mental stress and eating pattern on obesity gain. Furthermore, in most studies, mental stress status was determined by information collected on only one occasion, rather than on multiple occasions years apart.

In this study, we assessed each participant’s self-perception of mental stress, eating pattern, and related lifestyle factors by using questionnaires administered 5 years apart and investigated whether, over this 5-year period, the interaction between mental stress and eating pattern, defined as satiation eating or moderate eating, was associated with body mass gain in a population of Japanese working men without lifestyle-related diseases.

## METHODS

### Study population

The participants were members of a cohort study on cardiovascular disease that began in 1997 for local government employees in Aichi Prefecture, Japan aged 40 years or older. This cohort comprised 7414 persons (6114 men and 1300 women), 4003 of whom also participated in a self-administered questionnaire survey of lifestyle and disease history in 2002, among whom 2814 (2324 men and 490 women) provided written informed consent for the use of information obtained from the questionnaire and from health check-up data collected in 1997 and 2002. Among 2144 men with no missing data, 1064 men who had a history of cancer, heart disease, stroke, diabetes, hypertension, hyperlipidemia, or hyperuricemia were excluded so that any effects due to medication and intensive lifestyle modification on stress, BMI change, and lifestyle habits would be eliminated. After these exclusions, data from 1080 men remained for analysis. Data from all women were also excluded because there were too few women available for analysis. The study protocol was approved by the Ethics Review Committee of Nagoya University School of Medicine, Nagoya, Japan.

### Anthropometry

At the site of the annual health check-up, height and weight were measured to the nearest 0.1 cm and 0.1 kg, without shoes and with light clothing, and body mass index (BMI) was defined as weight in kilograms divided by the square of height in meters and was recorded every year. Change in BMI (ΔBMI) was determined by subtracting the value in 1997 from that recorded in 2002.

### Assessment of lifestyle factors and exposure to stress

Perception of mental stress was assessed by means of a self-administered questionnaire survey in 1997 and 2002. In response to the question, “Do you have much stress in your life?”, participants answered “very much”, “much,” “ordinary,” or “little”. Mental stress was defined as low if a participant answered “ordinary” or “little” in both years; for those who answered “very much” or “much” in at least 1 year, mental stress was defined as high. In a previous study, the validity of these responses was checked against questionnaires on job stressors and other lifestyle factors, including sleeping, exercise, and hours of overtime, and a satisfactory relation was confirmed.^23^^–^^25^

To determine the respondent’s eating pattern, participants were asked: “Do you eat to satiety?” The responses listed in 1997 were: “usually eat to satiety,” “usually eat moderately,” and “don’t know”. The responses were then dichotomized into “usually eat moderately” or “not,” which included the other 2 responses. The choices in 2002 were: “usually eat to satiety,” “tend to eat to satiety,” “tend to eat moderately,” and “usually eat moderately”. For our analysis, the first 2 and second 2 responses were divided to produce 2 dichotomous categories. Based on the combination of dichotomous answers in both years, participants were classified in the manner described above for mental stress as moderate eaters if they chose the second category response in both years or as satiation eaters if they chose the first category response in at least 1 year.

Frequency of physical exercise in each survey year was assessed based on participant agreement with one of the following statements: (1) “Not including job-related activities, I exercise once a month or more.” (2) “I exercise less than once a month or not at all.”

In both years, the choices for smoking status were: “do not smoke,” “have quit smoking,” and “smoke”. These were dichotomized into non-smoker (for the first 2 responses) and current smoker (for the third response). The items regarding alcohol were frequency and amount of ethanol per drink. This information was combined to produce an alcohol intake level of “none,” “less than 23 g/day of ethanol,” and “23 g/day or more,” as described in a previous report,^24^ and was dichotomized into those who drank less than 23 g/day and those who drank 23 g/day or more. Five-year exposure to each of these habits was assessed in the same manner as that used for mental stress. Statistical differences in these rates between the groups in question were examined by using the chi-square test.

### Other measurements considered

Daily energy intake (kcal/day), as well as intakes (g/day) of animal fat and sweets, was obtained individually by using a brief self-administered diet history questionnaire (BDHQ) in 2002 only. Responses were used to evaluate quantitatively the respondent’s eating pattern. The BDHQ is a 4-page, structured questionnaire previously developed by one of the authors.^26^^–^^29^ Estimates of dietary intake of 48 food and beverage items, energy, and 42 kinds of nutrients were calculated based on the Standard Tables of Food Composition in Japan.^30^ The reliability of the BDHQ itself,^26^ as well as the short-term test-retest reliability of eating habits,^31^ has been reported.

### Statistical analyses

To examine the effect of the interaction between mental stress and eating patterns on ΔBMI over a 5-year period, we performed 2-way analysis of variance (ANOVA) and covariance (ANCOVA) with 2 fixed factors, ie, mental stress and eating pattern, together with the interaction term between them, as well as covariates for adjustment in the ANCOVA. The items used as essential covariates were age, BMI at baseline (1997), and dichotomized 5-year exposure levels of alcohol, smoking, and exercise habits. In additional analysis, daily energy intake in 2002 was further adjusted to exclude the effect of its variation within and between groups.

Then, to examine the effects of mental stress and eating pattern on body mass, ΔBMI was compared between participants classified as moderate eaters and those classified as satiation eaters in the low and high mental stress strata, under conditions unadjusted and adjusted for the covariates listed above. Differences in mean age, BMI, and daily intake of energy, animal fat, and sweets between 2 groups were tested by using the *t*-test. Intakes of sweets were logarithmically converted for normalization. Differences in the proportions of participants with respective 5-year exposure levels of smoking, drinking, and exercise, and the association between mental stress and eating pattern were tested by using the chi-square test. The difference between mean ΔBMI and unity was tested using the paired *t*-test. All statistical analyses were performed with the SPSS statistical package, version 15.0. Statistical significance was defined as *P* < 0.05 for 2-sided testing.

## RESULTS

The characteristics of participants are shown in Table 1. The mean age (± SD) was 46.6 ± 4.1 years and mean BMI significantly increased by 0.22 ± 1.18 kg/m^2^, from 22.62 ± 2.50 kg/m^2^ in 1997 to 22.84 ± 2.58 kg/m^2^ in 2002 (*P* < 0.001, paired *t*-test). The proportion of participants with high mental stress was 61.9%, and 64.2% reported eating to satiety. In participants, mean intakes of energy, animal fat, and sweets were slightly lower than the mean per capita intakes (2235 kcal/day, 29.3 g/day, and 15.8 g/day, respectively) of males in the same age group in the National Health and Nutrition Survey of the same year. Although the proportion of satiation eaters in the high-stress stratum (65.6% [438/668]; Table 2) was slightly higher than that in the low-stress stratum (61.9% [255/412]), the rates did not significantly differ (*P* = 0.2, chi-square test), which indicates that eating pattern was not associated with mental stress in this population.

**Table 1.** Characteristics of participants (*N* = 1080)

Items	Mean (SD) or Number (%)
Age in 1997, Mean (SD), years	46.6 (4.1)
BMI 1997, Mean (SD), kg/m^2^	22.62 (2.50)
BMI 2002, Mean (SD), kg/m^2^	22.84 (2.58)*
ΔBMI, Mean (SD), kg/m^2^	0.22 (1.18)
Perceived high mental stress, Number (%)	668 (61.9%)
Satiation eater, Number (%)	693 (64.2%)
Low exerciser, Number (%)	306 (28.3%)
Nondrinker or light drinker, Number (%)	501 (46.4%)
Non-smoker, Number (%)	570 (52.8%)
Quantitative indices from BDHQ in 2002	
Energy intake, Mean (SD), kcal/day	2064 (679)
Animal fat intake, Mean (SD), g/day	28.5 (12.8)
Sweets intake, Mean (SD), g/day	13.5 (8.1)

SD: standard deviation; BMI: body mass index; BDHQ: brief dietary habit questionnaire

*: *P* < 0.001 by Student paired *t*-test, as compared to the value in 1997.

**Table 2.** Characteristics of eating groups stratified by level of perceived stress

	Low-stress stratum			High-stress stratum		
		
	Moderate eaters	Satiation eaters	*P* value	Moderate eaters	Satiation eaters	*P* value
	(*n* = 157)	(*n* = 255)		(*n* = 230)	(*n* = 438)	
Age 1997, Mean (SE), years	47.6 (0.3)	46.5 (0.3)	0.007	46.5 (0.3)	46.4 (0.2)	0.7
Low exerciser, Number (%)	42 (26.8%)	82 (32.2)	0.2	76 (33.0)	106 (24.2)	0.02
Nondrinker or light drinker, Number (%)	78 (49.7%)	116 (45.5)	0.4	111 (48.3)	196 (44.7)	0.4
Non smoker, Number (%)	78 (49.7%)	140 (54.9)	0.3	112 (48.7)	240 (54.8)	0.1
BMI 1997, Mean (SE), kg/m^2^	21.83 (0.19)	22.92 (0.16)	<0.001	21.78 (0.14)	23.17 (0.12)	<0.001
BMI 2002, Mean (SE), kg/m^2^	22.05 (0.19)	23.06 (0.16)	<0.001	21.90 (0.15)	23.50 (0.13)	<0.001
ΔBMI, Mean (SE), kg/m2	0.23 (0.09)	0.14 (0.08)	0.5	0.12 (0.07)	0.33 (0.06)	0.03
Adjusted ΔBMI-1*, Mean (SE), kg/m^2^	0.23 (0.09)	0.13 (0.08)	0.8	0.12 (0.07)	0.34 (0.06)	0.002
Adjusted ΔBMI-2^†^, Mean (SE), kg/m^2^	0.23 (0.09)	0.13 (0.08)	0.8	0.12 (0.07)	0.34 (0.06)	0.002

SE: standard error; *n*: number; BMI: body mass index

*: ΔBMI estimated by adjusting for essential covariates, ie, age, BMI in 1997, and exercise, drinking, and smoking habits

^†^: ΔBMI estimated by adjusting for essential covariates and energy intake in 2002

The effect of the interaction between mental stress and eating pattern on unadjusted ΔBMI approached significance on 2-way ANOVA (F = 3.76; degrees of freedom: 1, 1076; *P* = 0.053); however, the effect on ΔBMI after adjustment for essential factors (F = 4.90, *P* = 0.03) and after further adjustment for energy intake (F = 4.91, *P* = 0.03) were both significant on ANCOVA.

The ΔBMI and other characteristics of the moderate eaters and satiation eaters in each stratum of perceived stress are shown in Table 2. No group experienced a decrease in BMI during the 5-year period, and mean BMI gain (± SE) was largest among satiation eaters in the high-stress stratum (0.33 ± 0.06 kg/m^2^) and smallest among moderate eaters in the high-stress stratum (0.12 ± 0.07 kg/m^2^).

In the low-stress stratum, the mean age (± SE) of satiation eaters was significantly lower than that of moderate eaters (46.5 ± 0.3 vs 47.6 ± 0.3 years, respectively; *P* = 0.007), and BMI in both 1997 and 2002 was significantly higher among satiation eaters, as compared to moderate eaters (*P* < 0.001 for both 1997 and 2002). Mean ΔBMI, whether unadjusted or adjusted, and proportions of respondents with a given lifestyle characteristic, did not differ by eating pattern.

In the high-stress stratum, mean age and lifestyle characteristics did not significantly differ between moderate and satiation eaters, except that the latter exercised significantly less often (Table 2). Mean BMI in 1997 and in 2002 were significantly higher among satiation eaters, as compared to moderate eaters, as was the case in the low-stress stratum (*P* < 0.001 for both 1997 and 2002). ΔBMI was significantly greater among satiation eaters, as compared to moderate eaters, whether unadjusted (0.33 ± 0.06 vs 0.12 ± 0.07 kg/m^2^, respectively; *P* = 0.03), adjusted for essential covariates (0.34 ± 0.06 vs 0.12 ± 0.07 kg/m^2^, respectively; *P* = 0.002), or adjusted further for energy intake (0.34 ± 0.06 vs 0.12 ± 0.07 kg/m^2^, respectively; *P* = 0.002), which was not the case among the low-stress stratum.

The quantitative indices for eating patterns in 2002 are shown in Table 3. In an analysis with both strata combined, satiation eaters had significantly higher mean intakes of energy and sweets. When stratified by stress level, however, only mean energy intake, among the indices examined, was significantly higher among satiation eaters, as compared to moderate eaters (2123 ± 32 vs 2005 ± 45 kcal/day, respectively; *P* = 0.03), in the high-stress stratum. By contrast, among the low-stress stratum, only intake of sweets was significantly higher in satiation eaters than in moderate eaters (14.1 ± 1.2 vs 7.8 ± 1.2 g/day, respectively; *P* = 0.01); however, the difference in energy intake did approach statistical significance.

**Table 3.** Energy, animal fat, and sweets intakes in 2002

	Low-stress stratum			High-stress stratum			Unstratified		
			
	Moderate eaters	Satiation eaters	*P* value	Moderate eaters	Satiation eaters	*P* value	Moderate eaters	Satiation eaters	*P* value
	(*n* = 157)	(*n* = 255)		(*n* = 230)	(*n* = 438)		(*n* = 387)	(*n* = 693)	
Energy, Mean (SE), kcal/day	1964 (54)	2079 (42)	0.09	2005 (45)	2123 (32)	0.03	1988 (32)	2107 (27)	0.006
Animal fat, Mean (SE), g/day	29.0 (1.1)	28.1 (0.9)	0.5	27.9 (0.8)	29.0 (0.6)	0.3	28.3 (0.7)	28.7 (0.5)	0.7
Sweets, Mean (SE), g/day	7.8 (1.2)	14.1 (1.2)	0.01	13.4 (1.1)	16.1 (1.1)	0.2	10.8 (1.1)	15.3 (1.1)	0.008

SE: standard error

## DISCUSSION

The mean BMI of this population increased over a 5-year period, as it did in the general Japanese population during the same period, as indicated by a series of National Health and Nutrition Surveys.^32^ This suggests that the increase in body weight was not particular to the present study population. Stress was not related to eating pattern in this healthy male population; thus, the pattern of stress-induced eating that has been observed in women^8^ was not seen.

In the present analysis, perception of mental stress and eating pattern over a 5-year period had a statistically significant interaction effect on BMI gain during that period, ie, satiation eating was associated with a significantly higher BMI gain than moderate eating among participants who were highly stressed; this association was not observed in those who were less stressed. These results remained unchanged even after ΔBMI was adjusted for age, lifestyle factors, and BMI at baseline.

In animal experiments, stress resulted in an increase in either,^19^ or both,^13^^,^^14^^,^^18^ energy intake and weight, or a reduction in body weight,^15^^–^^17^ depending on the nature and duration of the stress (acute or chronic), diet, and species. Chronic stress caused by isolation housing, however, has been consistently shown to increase both eating and weight.^8^ Meanwhile, in humans, the association between chronic stress and BMI was independent and positive,^4^^,^^7^ inverse,^9^ or absent.^3^^,^^5^^,^^6^ Thus, the effect of chronic stress on eating habits and weight remains controversial. Gender differences in stress-induced eating^8^ may partly explain this disagreement. The effect of an interaction between eating pattern and stress on long-term ΔBMI, which has not been previously examined, but was observed in the present study, might also be a factor.

In most studies on humans, chronic mental stress was assessed by using data obtained from one investigation rather than from successive observations. In the present study, data on lifestyle factors were obtained on two occasions 5 years apart, with the exception of energy and food intakes. Hence, the association between lifestyle and ΔBMI over this period should reflect long-term phenomena better than data based on just one data collection, as noted in the study of the association between chronic stress and metabolic syndrome by Chandola et al.^33^

Among highly stressed participants, energy intake was significantly higher in satiation eaters than in moderate eaters, and this difference in energy intake might have contributed to the difference in BMI gain between these groups. However, this is not consistent with the absence of a difference in ΔBMI between these 2 groups in the low-stress stratum, where the difference in energy intake was of borderline significance and nearly the same as that observed in the high-stress stratum. Moreover, the presence of a significant interaction after adjustment for energy intake suggests that some mechanism other than simple differences in calorie intake and the lifestyle factors examined in the present study might better explain the variation in ΔBMI.

Although elucidating the mechanism of interaction is beyond the scope of this article, recent animal experiments indicate that the caloric efficiency of high energy food, with respect to weight gain, was higher in stressed rats than in control rats,^18^^,^^22^ and that stress enhanced weight gain in genetically predisposed rats that were kept on a high-energy diet.^18^ Furthermore, in stressed, as compared to unstressed, rats a larger proportion of energy intake was attributable to consumption of comfort food.^22^ Stressed humans also consumed more high-fat foods.^5^^,^^10^ In the present study, the intake of sweets among the satiation eaters exceeded that of the moderate eaters in the stratum with low stress; however, this tendency was less apparent among those with high stress, a finding that conflicts with those of previous reports.^5^^,^^10^ When this result is considered together with the finding that a significantly greater gain in BMI was seen only in the high-stress stratum, despite the fact that the energy increase related to satiation eating was almost the same in the high- and low-stress strata, it appears that caloric efficiency is somehow increased by stress, and that this mechanism might be the basis of the interaction.

### Limitations

This study is subject to the following limitations. Because the present results were obtained in a population with a homogeneous social environment (ie, public servants) and no lifestyle-related disease, the results thus obtained may not necessarily be applicable to the general population. However, any associations observed would be less likely to have been affected by confounders such as medication use and intensive lifestyle modifications.

Second, although so-called stress-induced eating is reported to be more prevalent in women than in men,^8^ gender differences could not be examined in the present study due to the small number of potential female participants.

Third, although all data on lifestyle factors were collected twice, 5 years apart, quantitative data on energy and food intake were obtained in 2002 only. Because participants with lifestyle-related disease were not included in the analysis, we assumed that energy and food intakes in 2002 were the same as those at baseline (1997). However, a long-term association between change in BMI and energy or food intakes might have been less clear than an association between change in BMI and lifestyle factors, which were assessed twice.

Fourth, the true interaction effect might have been distorted by the coexistence of participants who increased or decreased their exposure to the 2 factors of concern and those who maintained a high exposure. This distorting effect needs to be examined in a larger sample, so as to confirm the interaction between exposures to more refined factors.

Lastly, although we gathered data longitudinally over a 5-year period, the analysis itself was cross-sectional. Thus, a cause-effect relationship cannot be ascertained. To take one example from the present study, the group that had the greatest increase in BMI had the highest mean BMI at baseline, in accordance with the results of a recent study^34^ of the effect of stress on weight gain in women. Although we adjusted for the difference in BMI in 1997 in our analysis of the effect on ΔBMI, it can still be argued that obesity itself may have been responsible for the greater BMI gain, higher mental stress, and satiation eating. A well-designed longitudinal study to confirm these associations is warranted, in order to examine their applicability and underlying mechanisms in a population that is either older or has a higher prevalence of lifestyle-related diseases.

In conclusion, satiation eaters showed a significantly greater increase in body mass than did moderate eaters only under high-level mental stress, independently of other lifestyle habits as well as energy intake. These findings indicate the presence of an interaction between eating pattern and long-term mental stress in middle-aged healthy male workers.
