# Effect of the Interaction between Mental Stress and Eating Pattern on Body Mass Index Gain in Healthy Japanese Male Workers

**DOI:** 10.2188/jea.JE20080066E

**Published:** 2009-09-05

**Authors:** Hideaki Toyoshima, Nobutaka Masuoka, Shuji Hashimoto, Rei Otsuka, Satoshi Sasaki, Koji Tamakoshi, Hiroshi Yatsuya

In the report, the second sentence of the Abstract should read as follows: “A population of 1080 healthy Japanese male local government employees without lifestyle-related diseases **at baseline** were studied.”

In addition, the third sentence of the Methods section should read as follows: “Among 2144 men with no missing data, **1045 men who had a history of heart disease, stroke, diabetes, hypertension, hyperlipidemia, or hyperuricemia in 1997 and 19 men who had a cancer history in 1997 or 2002** were excluded….”

The authors apologize for these errors.

