# Effect of the Interaction between Mental Stress and Eating Pattern on Body Mass Index Gain in Healthy Japanese Male Workers

**DOI:** 10.2188/jea.JE20110067

**Published:** 2011-09-05

**Authors:** Hideaki Toyoshima, Nobutaka Masuoka, Shuji Hashimoto, Rei Otsuka, Satoshi Sasaki, Koji Tamakoshi, Hiroshi Yatsuya

There were numerical errors in the report when the article was printed.

First, the mean and SE values in the third sentence in the Result section of the Abstract should read as follows: “adjusted mean [SE]: 0.37 ± 0.06 kg/m^2^ vs. 0.04 ± 0.08 kg/m^2^, *P* = 0.002.”

Second, the last 4 sets of mean and SE values in the fifth paragraph of the Results (page 91) should read as follows: “adjusted for essential covariates (0.37 ± 0.06 vs 0.04 ± 0.08 kg/m^2^, respectively; *P* = 0.002), or adjusted further for energy intake (0.37 ± 0.06 vs 0.05 ± 0.08 kg/m^2^, respectively; *P* = 0.002) …”

Third, the adjusted mean (SE) values for ΔBMI-1 and ΔBMI-2 in Table 2 should read as follows (from left to right): 0.16 (0.09), 0.18 (0.07), 0.04 (0.08), 0.37 (0.06); and 0.16 (0.09), 0.18 (0.07), 0.05 (0.08), 0.37 (0.06), respectively. No *P*-values in Table 2 need to be changed. These corrections do not alter the main findings or the implications of those findings. The authors apologize for these errors.

